# Trichomes form genotype-specific microbial hotspots in the phyllosphere of tomato

**DOI:** 10.1186/s40793-020-00364-9

**Published:** 2020-09-17

**Authors:** Peter Kusstatscher, Wisnu Adi Wicaksono, Alessandro Bergna, Tomislav Cernava, Nick Bergau, Alain Tissier, Bettina Hause, Gabriele Berg

**Affiliations:** 1 Institute of Environmental Biotechnology, Graz University of Technology, Graz, Austria; 2 Austrian Centre of Industrial Biotechnology (ACIB GmbH), Graz, Austria; 3 Department of Cell and Metabolic Biology, Leibniz Institute of Plant Biochemistry, Halle, Germany

**Keywords:** Plant microbiome, Bacterial communities, Plant-microbe interactions, *Solanum habrochaites*, *Solanum lycopersicum*, Plant microhabitat, Phyllosphere, Trichomes

## Abstract

**Background:**

The plant phyllosphere is a well-studied habitat characterized by low nutrient availability and high community dynamics. In contrast, plant trichomes, known for their production of a large number of metabolites, are a yet unexplored habitat for microbes. We analyzed the phyllosphere as well as trichomes of two tomato genotypes (*Solanum lycopersicum* LA4024, *S. habrochaites* LA1777) by targeting bacterial 16S rRNA gene fragments.

**Results:**

Leaves, leaves without trichomes, and trichomes alone harbored similar abundances of bacteria (10^8^–10^9^ 16S rRNA gene copy numbers per gram of sample). In contrast, bacterial diversity was found significantly increased in trichome samples (Shannon index: 4.4 vs. 2.5). Moreover, the community composition was significantly different when assessed with beta diversity analysis and corresponding statistical tests. At the bacterial class level, *Alphaproteobacteria* (23.6%) were significantly increased, whereas *Bacilli* (8.6%) were decreased in trichomes. The bacterial family *Sphingomonadacea* (8.4%) was identified as the most prominent, trichome-specific feature; *Burkholderiaceae* and *Actinobacteriaceae* showed similar patterns. Moreover, *Sphingomonas* was identified as a central element in the core microbiome of trichome samples, while distinct low-abundant bacterial families including *Hymenobacteraceae* and *Alicyclobacillaceae* were exclusively found in trichome samples. Niche preferences were statistically significant for both genotypes and genotype-specific enrichments were further observed.

**Conclusion:**

Our results provide first evidence of a highly specific trichome microbiome in tomato and show the importance of micro-niches for the structure of bacterial communities on leaves. These findings provide further clues for breeding, plant pathology and protection as well as so far unexplored natural pathogen defense strategies.

## Background

Plants can be considered as holobionts that are embedded in complex microbial interaction networks, and microorganisms contribute significantly to host’s health and fitness [1]. While plant-microbe interactions and their importance was extensively studied in the rhizosphere, interactions in the plant phyllosphere are currently less explored [2, 3]. The phyllosphere with a global leaf area of about 10^9^ km^2^, which is bigger than the planet’s surface, provides one of the largest habitats for microorganisms [4]. Many microorganisms including filamentous fungi, yeast, archaea and algae were found to colonize leaves [5]. So far, the plant phyllosphere was found to be influenced by a number of different parameters including the plant genotype but also environmental fluctuations and macro and micro climatic conditions [6–8]. Moreover, the importance of the phyllosphere inhabiting microbiota in the defense of plants against pathogens was shown [8, 9]. The *Arabidopsis* phyllosphere was characterized by a high degree of spatial differences, indicating that the colonization of leaves by microorganisms is not uniform [10]. However, their spatial phyllosphere distribution is still not entirely resolved, and especially the colonization of leaf niches formed by epidermal outgrowths, e.g. hairs or trichomes, is not yet understood [11].

Glandular trichomes are known to occur in multiple plant families, including *Asteraceae*, *Lamiaceae* and *Solanaceae* and come in multiple shapes and functions [12–14]. They are characterized by a high production of secondary metabolites, which are not only relevant for plant communication, but also of high value for industrial exploitation [12]. The prevalent chemical classes produced in trichomes include alkaloids, flavonoids, short branched-chain acyl sugars, phenolics and isoprenoids [15–17]. Plant glandular trichomes are able to secrete and store large amounts of volatile organic compounds (VOCs) but mechanisms allowing transport of VOCs to the cavity, however, preventing their diffusion are not known [18]. It was previously shown that different tomato genotypes have different metabolite spectra in their glandular trichomes [13, 19]. Additionally, trichomes were suggested as infection sites for pathogenic bacteria and fungi, which was observed using different microscopic techniques [20]. Other studies using the pathosystem *Clavibacter michiganensis* – tomato confirm trichomes as infection sites [21]. Interestingly, distinct wild tomato species, including *S. habrochaites* LA2128, were tolerant to *C. michiganensis*, although the mechanism underlying resistance remains unclear [22]. Scanning electron micrographs of leaves and isolation studies already indicated trichome surfaces as preferential microbial niche [23, 24] but currently there are no microbiome data to evidence that. Recently, it was shown for the tomato rhizosphere how plants shape their endophytic microbiome due to the biosynthesis of phytohormones [25]. We therefore hypothesized that due to their specific chemical composition, glandular trichomes may form a specific microbiome distinct from that of the leaf.

In the present study, we focused on the analysis of microbiomes associated with tomato trichomes. In a deepening approach, we have assessed the differences in the microbiome of various plant phyllosphere microhabitats by differentiation between microbial communities on trichome-free leaf surfaces and trichomes themselves. Therefore, we used leaves of two different tomato genotypes that are characterized by different structures and densities of trichomes, namely *Solanum lycopersicum* LA4024 (also known as E6203) and the wild tomato *Solanum habrochaites* LA1777. Tomato species have a complex set of trichomes with three types of non-glandular trichomes (II, III, V) and four types of glandular trichomes (I, IV, VI and VII) [26]. As in most *S. lycopersicum* lines, the most abundant type of glandular trichomes on the leaves and stems are the type VI; the major metabolites produced by these trichomes are mono- and sesquiterpenes [27, 28]. In *S. habrochaites* LA1777, type VI trichomes produce sesquiterpene carboxylic acids which can represent up to 15% of the leaf dry weight [29–31]. The type VI trichomes of *S. habrochaites* have a different shape and a much larger storage cavity, which in part explains the higher amounts of terpenes accumulated in that species [12, 32, 33]. Type IV trichome produce acylsugars which are secreted and result in a sticky layer where insects can be trapped [16, 34–36]. Based on these differences, we thus hypothesize that the trichome microbiota of both tomato genotypes is highly different.

To our knowledge this is the first study investigating the microbiome structure of plant trichomes applying high-throughput sequencing technologies. More remarkably, this study investigates the smallest plant microhabitat ever. It provides the basic knowledge related to spatial differences in microbial community composition between plant microhabitats and expands our understanding of plant phyllosphere microbiomes.

## Methods

### Description of plant material

The tomato lines used for the leaf and trichome microbiome experiments were *Solanum lycopersicum* LA4024 (also known as E6203) and the wild tomato *Solanum habrochaites* LA1777 both obtained from the Tomato Genetics Resource Center at UC Davis (TGRC). LA4024 carries the mutations *obscuravenosa* (*obv*), *self-pruning* (*sp*) and *uniform ripening* (*u*) (https://tgrc.ucdavis.edu/Data/Acc/AccDetail.aspx?AccessionNum=LA4024). The *obv* mutation leads to green veins, the *sp* to a determinate growth habit and *u* to a uniform green fruit color. LA4024 has been used as a parent to generate a set of introgression lines from a cross with *S. habrochaites* LA1777 [37].

LA1777 is an accession of the wild tomato *S. habrochaites* that was collected at Rio Casma in Peru (full description available at https://tgrc.ucdavis.edu/Data/Acc/dataframe.aspx?start=AccSearch.aspx&navstart=nav.html). This is a self-incompatible line, which was propagated vegetatively in the greenhouses of the Leibniz Institute of Plant Biochemistry. LA1777 has a much higher density of glandular trichomes on the surface of its leaves and stems.

### Sample collection and DNA extraction

The two above mentioned tomato lines were used to obtain mature plants for phyllosphere microbiome analyses. Plants were grown in the greenhouse in sterilized soil and trichomes were collected as described in [12, 38]. Briefly, young leaves were brushed with a paint brush, previously dipped in liquid nitrogen, to detach trichomes from young leaves. The trichomes were collected in a mortar filled with liquid nitrogen. The samples were further sieved to remove plant debris and transferred to tubes and stored at − 80 °C before processing (Additional file 1). Leaves for trichome removal were collected from 15 or 50 plants per replicate for LA1777 and LA4024, respectively. Brushed leaves without trichomes and unbrushed leaves (Fig. 2a-d) were collected as well. A total of 0.01 g of trichomes/sample were pelleted with 5 mL sodium chloride solution (0.85%). Similarly, 0.1 g of leaf samples were homogenized in 50 mL sodium chloride solution and 5 mL were pelleted. Sample pellets were used for DNA extraction. Total DNA was extracted using the FastDNA SPIN Kit for Soil and the FastPrep Instrument (MP Biomedicals, Santa Ana, CA, USA) according to the manufacturer’s protocol. DNA samples were quality checked using a Nanodrop 2000 (Thermo Scientific, Wilmington, DE, USA) and stored at − 20 °C for further PCR reactions.

### Microscopic in situ visualization of bacteria on trichomes

Bacteria on leaf sections from both cultivars were fixed with 4% paraformaldehyde/phosphate-buffered saline at 4 °C over-night. Subsequently, the samples were stained with the LIVE/DEAD™ BacLight™ Bacterial Viability Kit (Molecular Probes). The imaging was performed using a confocal laser scanning microscope (Leica TCS SPE confocal microscope, Leica Microsystems). Excitation wavelengths of 488 and 532 nm were used for the SYTO® 9 and propidium iodide fluorescent dye, respectively. The light emission was detected in the range of 496–560 nm for SYTO® 9 and 600–680 nm for propidium iodide. Settings for photomultiplier gain and offset were adjusted to obtain an optimal signal/noise ratio. The confocal stacks were merged to obtain a maximum projection of all channels.

### Quantitative real time PCR

Total DNA extracts from the samples were further used for quantification of specific genes using qPCR. Total 16S rRNA gene copy numbers were obtained using the Unibac II 515f/927rP primer pair (515f: 5′-GTG CCA GCA GCC GC-´3 and Unibac-II-927rP: 5′-CCC GTC AAT TYM TTT GAG TT-´3) [39]. The quantification was performed with a Corbett Research TM thermocycler (Rotor-Gene 6000, Corbett Research, United Kingdom) and SYBR Green PCR master mix TM (KAPA Biosystems, USA). The standard regression curve was obtained using a *Bacillus cereus* 16S rRNA gene fragment and further 1:10 dilutions. Three replicates of each standard dilution were prepared to generate a mean value. The standard regression curve was employed to determine the gene copy numbers in the analyzed samples and numbers were normalized to the weight of initial sample. All PCR reactions were performed in triplicates.

### Amplification of 16S rRNA gene fragments

Isolated DNA from the two tomato genotypes and three sample types (each in 4 replicates) were used for amplification of the 16S rRNA gene V4 and V5 hypervariable region using the 515f/926r primer pair (515f: 5′-GTGYCAGCMGCCGCGGTAA-3′; 926r: 5′ CCGYCAATTYMTTTRAGTTT-3′) [40]. All PCR reactions were performed in triplicates. The PCR mix was amplified in 35 cycles at 94 °C denaturation for 45 s, 50 °C annealing for 60 s and 72 °C elongation for 90 s. Barcode sequences for multiplexing of the data were used as provided by the earth microbiome project (earthmicrobiome.org). In addition, peptide nucleic acid (PNA) PCR clamps were used to block the amplification of plastid and mitochondrial 16S rRNA gene sequences of plants during the PCR amplification. The amplicons were purified using the Wizard SV Gel and PCR Clean-Up System (Promega, Madison, WI) and pooled in equimolar concentrations. The barcoded Illumina library was sent for paired-end Illumina MiSeq sequencing (GATC Biotech, Berlin, Germany). The 16S rRNA gene fragment raw reads obtained from the sequencing company were deposited at the European Nucleotide Archive (ENA) under the project number PRJEB37893.

### Bioinformatic analysis

Paired-end reads were quality checked and demultiplexed using cutadapt [41]. Only forward reads were used due to low quality of reverse reads. Bioinformatic analysis for amplicon sequencing analysis was performed using the open-source QIIME2 version 2018.4.0 pipeline (https://qiime2.org). Primer sequences were removed and the DADA2 algorithm in QIIME2 was used to quality filter, denoise and remove chimeric sequences [42] resulting representative sequences, called amplicon sequences variants (ASVs), and a feature table. ASVs were classified using the vsearch algorithm and the SILVA v132 database [43, 44].

### Statistical analysis

The R version (R Core Team) was used to perform statistical analysis and create graphs unless stated otherwise. Significant differences (*p* < 0.05) of bacterial gene copy numbers per gram of samples were analyzed using the Kruskal Wallis test. Resulted ASV tables and taxonomic classifications were uploaded into R via phyloseq [45]. Bacterial analysis was performed by rarefying the dataset to the lowest number of read counts by randomly selecting subsets of sequences. Plot bars were used to visualize taxonomic composition. Differences in alpha bacterial diversity were assessed using the Kruskal Wallis test followed by pairwise comparison at *P* < 0.05. The beta diversity based on a normalized Bray-Curtis dissimilarity matrix was generated and then subjected to permutational analysis of variance (PERMANOVA, 999 permutations) to test for significant effects of sample type and tomato genotype on the bacterial community structures. The distance matrices were visualized by using a non-metric multidimensional scaling (NMDS) plot. Finally, the DESeq2 method [46] was performed to determine the differences between the taxa abundances in different sample type. ASVs were defined significantly different if the adjusted *P* value (Benjamini-Hochberg adjustment) was less than 0.05.

## Results

### Quantification of bacterial population density in tomato plants

To determine the bacterial abundance in the phyllosphere microhabitats targeted real-time quantitative PCR (qPCR) was applied. The qPCR results showed that bacterial density varied between 5.24 × 10^8^ and 6.94 × 10^9^ 16S rRNA gene copy numbers per gram of sample. However, statistical significance tested using the Kruskal-Wallis test indicated that there was no effect of sample type (*P* = 0.356) and tomato genotype (*P* = 0.158) on bacterial density (Additional File 2).

### In situ visualization of bacteria on tomato trichomes

**Fig. 1:**
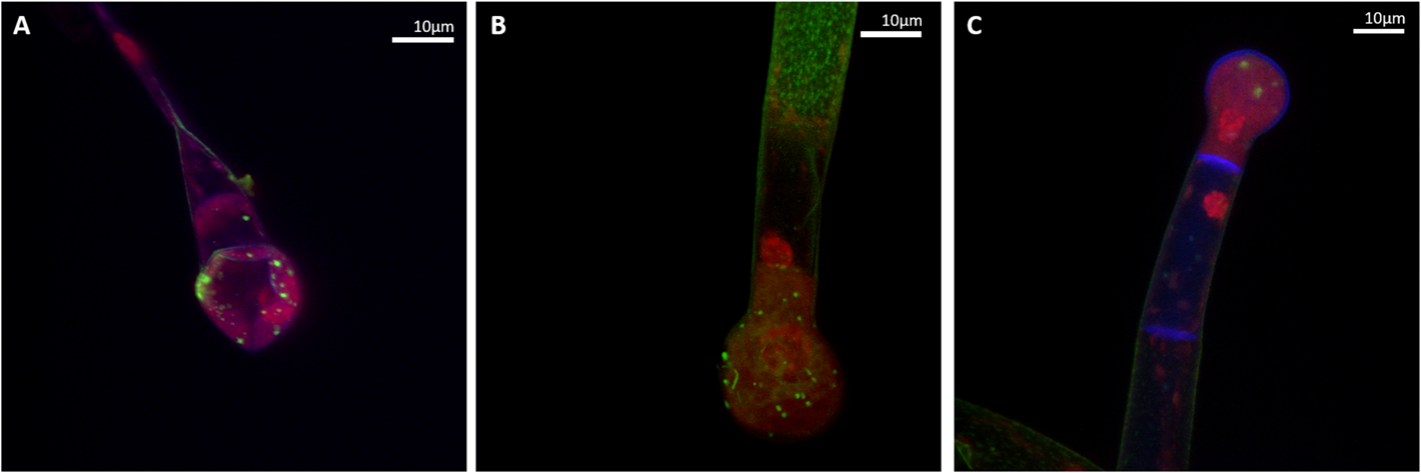
Micrographs showing trichomes of genotype LA1777 (**a**) and LA4024 (**b** & **c**) with living bacteria colored in green (bright spots). The visualization was conducted with confocal laser scanning microscopy (CLSM) in combination with the LIVE/DEAD™ BacLight™ Bacterial Viability Kit. Bacteria are accumulated in close proximity to trichome heads in all analyzed samples and were alive at the time point of sample collection as indicated by differential staining. Propidium iodide intercalates with plant DNA, thus the plant nuclei are stained in red

Confocal laser scanning microscopy (CLSM) was implemented in combination with differential DEAD/LIVE staining to visualize microbes colonizing different trichome types (Fig. 1a - c). Bacteria were found on type IV (Fig. 1a) as well as type I (Fig. 1b & c) trichomes. The colonization density of the detected bacteria was similar among samples irrespective of the trichome type. Moreover, the micrographs display a diverse bacterial community as indicated by cell morphology. The visualized bacteria were located in close proximity to the trichome head from both genotypes (Fig. 1a - c).

### General assessment of amplicon libraries

Composition of bacterial communities were obtained using amplicon sequencing. After quality filtering of the raw reads and exclusion of non-target sequences (mitochondria and chloroplasts), a total of 102,722 high quality reads from bacterial libraries were retained with 442 to 15,788 reads per sample which were assigned to 1421 bacterial amplicon sequences variants (ASVs). Even though a high proportion of reads were assigned to non-target taxa that were detected due to the nature of the samples (high occurrence of chloroplasts and mitochondria), the average ASV number found in the sample was 69.2 (Additional file 3). Interestingly, trichome samples had a relatively lower proportion of non-target reads in comparison to other samples.

### Observed alpha and beta diversity of bacterial communities

With respect to sample type, the Kruskal-Wallis test showed that bacterial richness is significantly higher in trichomes compared to other sample types (*P* < 0.001). A higher bacterial richness indicated by the Shannon index (H′) was observed in trichomes samples (H′ = 4.4) when compared to leaves (H′ = 2.5) and leaves without trichomes (H′ = 2.8). No significant differences were observed between leaves and leaves without trichomes. With respect to tomato genotype, samples from LA1777 had a relatively higher alpha bacterial diversity (H′ = 3.4) compared to LA4024 (H′ = 3.0). However, Kruskal-Wallis test showed that the tomato genotype did not influence bacterial richness according to number of ASV and Shannon diversity index (*P* = 0.157 and *P* = 0.248, respectively) (Additional file 3, Fig. 2e).

**Fig. 2:**
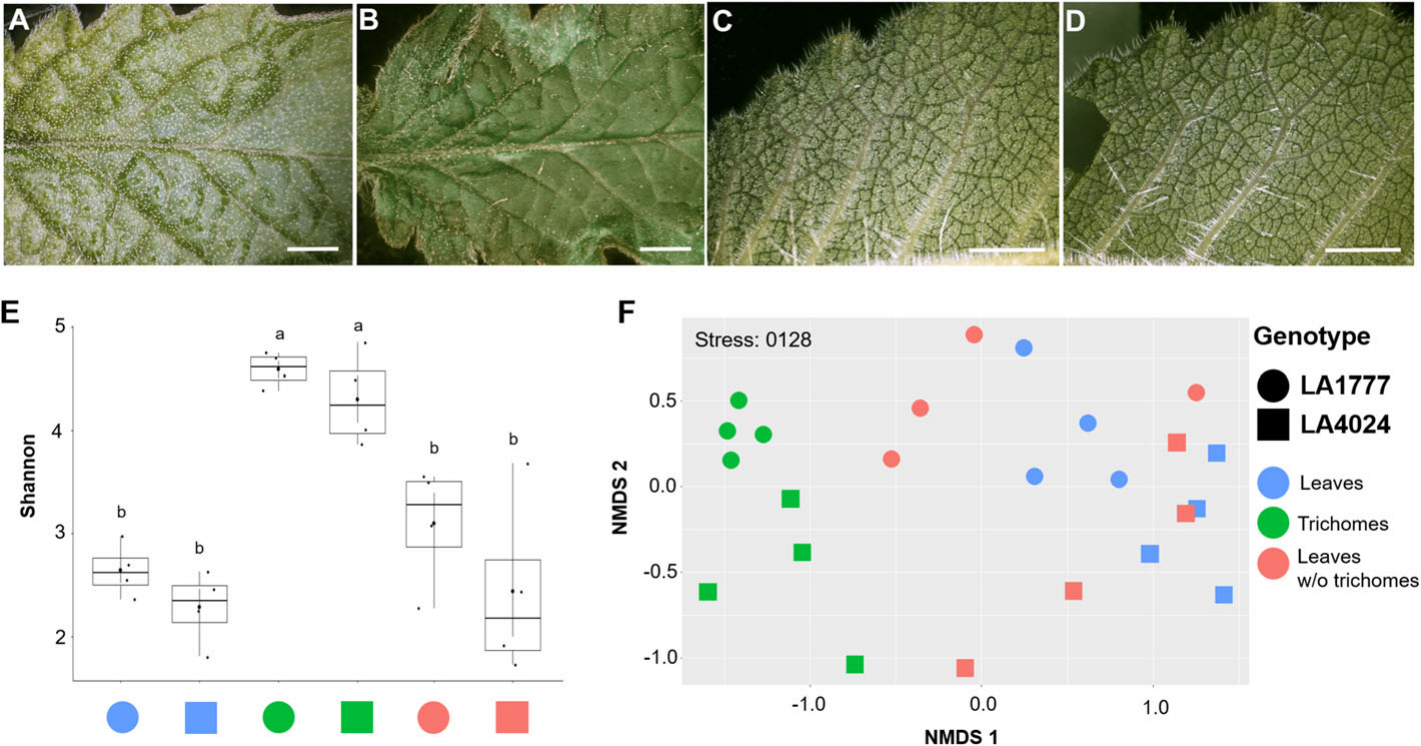
Micrographs of tomato leaves (**a**-**d**) as well as observed alpha (**e**) and beta (**f**) diversity of tomato-associated bacterial communities. Leaf of *Solanum lycopersicum* LA4024 before (**a**) and after (**b**) brushing to isolate trichomes. Leaf of *S. habrochaites* LA1777 before (**c**) and after (**d**) brushing to isolate trichomes. Note that glandular trichomes are visible as white dots in (**a**) and (**c**) before they have been removed by brushing. All scalebars represent 5 mm. The bacterial richness was assessed with the Shannon diversity index (**e**). Different sample types and tomato lines were separately assessed and the significantly differences tested by Kruskal-Wallis (indicated by letters). Beta diversity was accessed by visualizing a Bray Curtis distance matrix in a non-metric multidimensional scaling (NMDS) plot showing bacterial differences between each sample (**f**). Shapes represent tomato genotypes (circle: LA1777, square: LA4024); colors represent sample type (blue: Leaves, green: Trichomes, and red: brushed leaves without trichomes)

Observed beta diversity visualized using non-metric multidimensional scaling (NMDS) showed discrete clusters according to sample type as well as tomato genotype. These factors influenced the bacterial community structures significantly (*P* < 0.05). Sample type was shown to be the dominant factor that influenced the bacterial variation (23.9%) whereas tomato genotype only explained 8.4% of the variation. Trichome samples tend to cluster closer together whereas leaves with and without trichomes formed another cluster. Despite only a small variation that was statistically explained by tomato genotype, a clear separation of the two genotypes was observed in all sample types (Fig. 2f).

### Identification of microbial taxa in different microhabitats

**Fig. 3:**
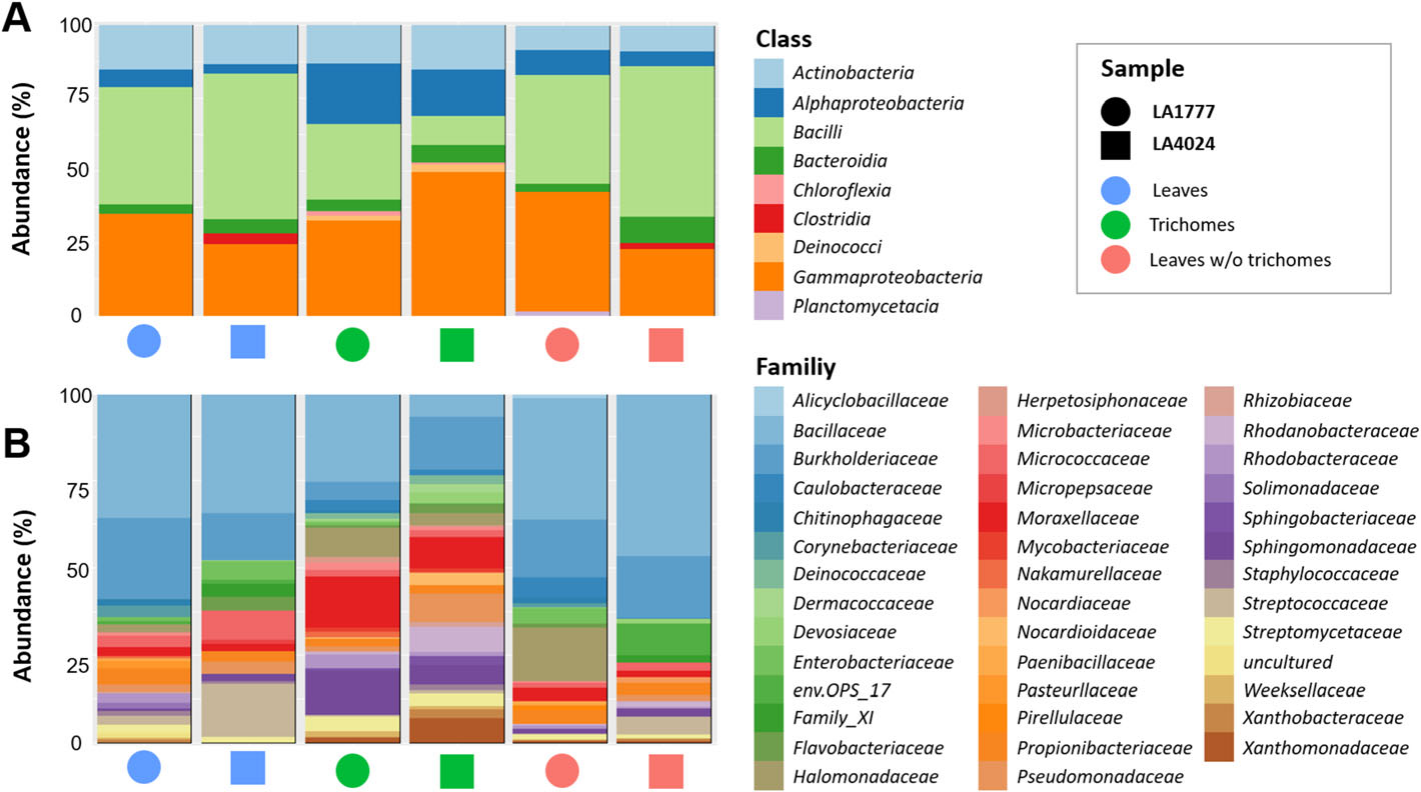
Relative abundance plot of the top 100 most abundant bacterial ASVs at class (**a**) and family (**b**) level. Different tissue types and tomato lineages were assessed separately. Shapes represent tomato genotypes (circle: LA1777, square: LA4024); colors represent sample type (blue: Leaves, green: Trichomes, and red: brushed leaves without trichomes)

Differences in the taxonomic composition were visualized by assessing the 100 most abundant bacterial ASVs (Fig. 3). The bacterial communities present in all samples showed a high proportion of *Proteobacteria* (44.4%) and *Firmicutes* (30.8%). Comparing individual sample types, no substantial differences were found on phylum level. The analysis on class level, however, showed that the bacterial classes *Bacilli* (39.1%) and *Gammaproteobacteria* (29.5%) were the most abundant bacterial class in leaves with and without trichomes, whereas in trichome samples, a higher relative abundance of *Alphaproteobacteria* (23.6%) was observed for both genotypes (Fig. 3a). At family level, the most abundant families were *Bacillaceae* and *Burkholderiaceae* that accounted for 38.3% relative abundance regardless of the sample type. In trichome samples, a relatively higher number of bacterial families were generally observed compared to the others. This result supported the higher bacterial diversity generally found in trichomes in comparison to other samples types. Especially the relative abundance of *Moraxellacea* (6.4%) and *Sphingomonadacea* (8.4%) were higher in trichomes compared to the other sample types. The family of *Bacillaceae* (8.6%), which had a relatively high abundance in leaves, showed the opposite pattern (Fig. 3b). Moreover, we observed low abundant bacterial families (relative abundance > 0.1%) i.e. *Hymenobacteraceae* and *Alicyclobacillaceae* that were found exclusively in trichomes.

**Fig. 4:**
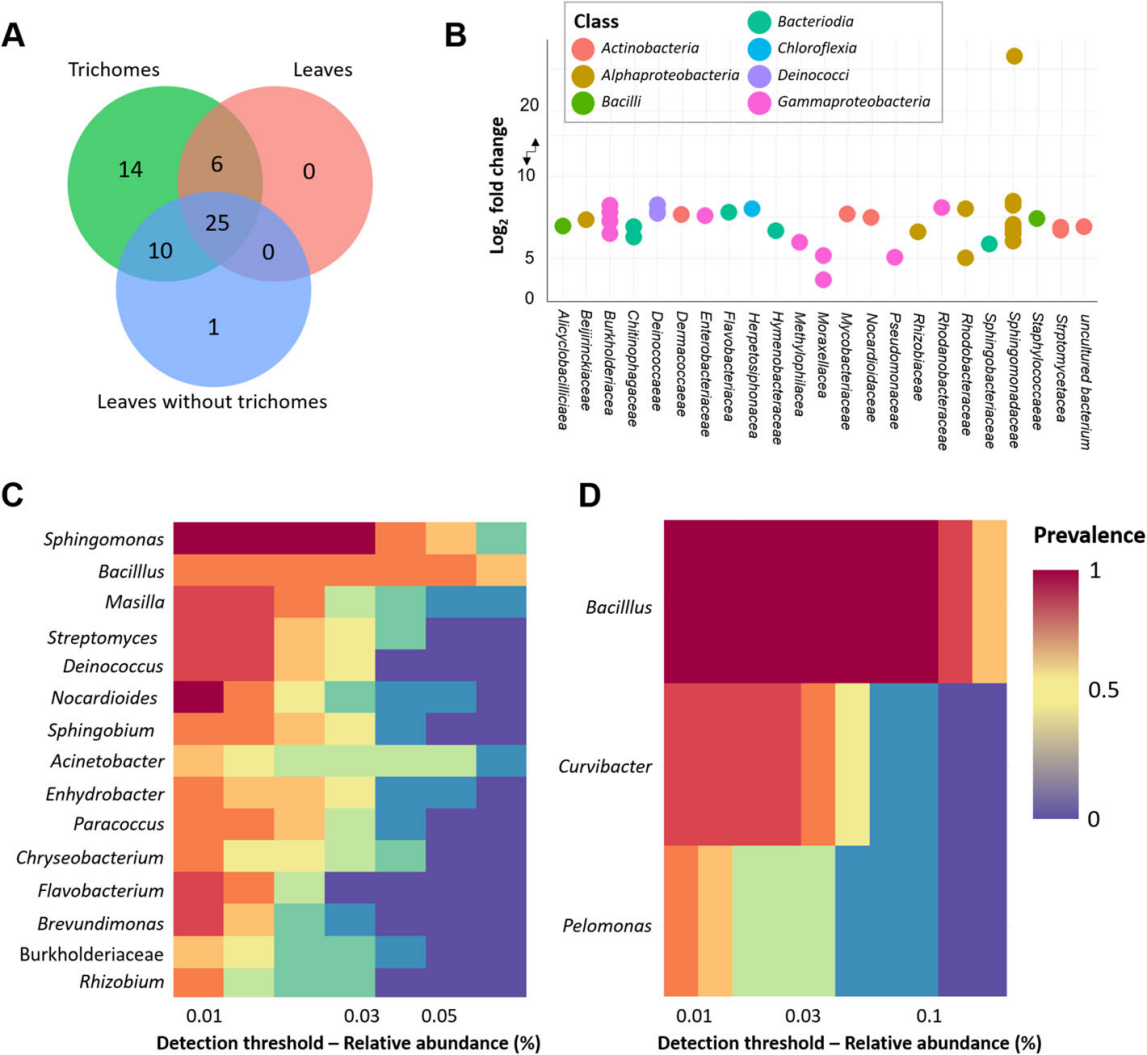
Analysis of bacterial core microbiomes. Unique and shared ASVs are shown in a Venn diagram (**a**). Bacterial ASVs that are significantly increased in trichomes compared to leaves without trichomes were determined by DESeq2 analysis (**b**). Core microbiomes of trichomes (**c**) and leaves without trichomes (**d**) are shown in a heatmap

Trichome samples that were shown to harbor a higher bacterial diversity compared to the other two sample types, overall also harbored the highest number of ASVs. In total, 25 ASVs were shared between all samples. Trichome samples in contrast harbored 14 ASVs which were exclusively found in those samples (Fig. 4a). DESeq2 analysis was performed to investigate which taxa were specifically increased in trichomes in comparison to leaves without trichomes. A total of 20 ASVs which were dominated by *Burkholderiaceae* (*n* = 4) and *Sphingomonadaceae* (*n* = 7) families respectively were significantly increased (Fig. 4b). Six ASVs from the *Actinobacteria* also showed the same pattern. The analysis of core microbiomes found in the different sample types showed a highly more diverse core microbiome in trichome samples. Especially the genus *Sphingomonas*, a member of the family *Alphaproteobacteria*, was identified as a central element in the core microbiome of trichome samples but was not found in the core of leaves without trichomes (Fig. 4c & d). A core microbiome was identified in each genotype; however, higher abundance of *Actinobacteria* and *Proteobacteria* ASVs were found to be characteristic for trichome samples.

### Genotype specific enrichment of trichome-colonizing microorganisms

**Fig. 5:**
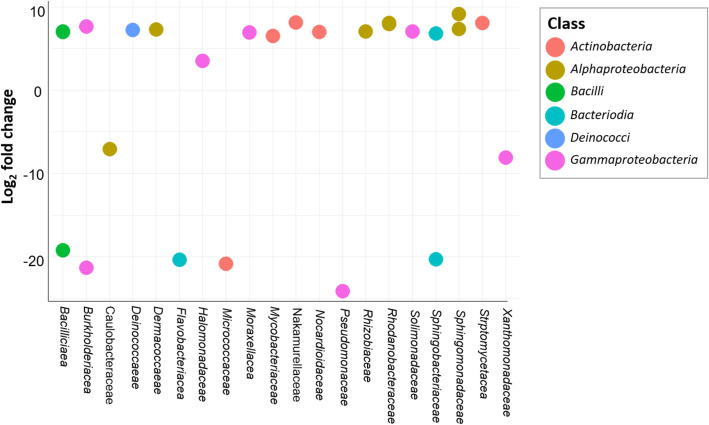
Microbial differences in trichome microbiomes. Bacterial ASVs that are significantly differential abundant in trichomes from *S. habrochaites* LA1777 compared to trichomes from *S. lycopersicum* LA4024 were determined by DESeq2 analysis. Log_2_ fold change of ASVs color coded based on class is shown

Overall, trichome samples were found to harbor increased microbial diversities, however, genotype specific differences were also observed. Compared to *S. lycopersicum* LA4024, trichomes of *S. habrochaites* LA1777 carried a higher abundance of *Bacilli*, but a lower abundance of *Gammaproteobacteria*. At family level, increased levels of *Bacillaceae*, *Moraxellaceae* and *Sphingomonadaceae*, as well as decreased abundances of *Burkholderiaceae*, *Pseudomonadaceae* and *Xanthomonadaceae* were observed (Fig. 3). Significance of taxonomic differences was further assessed using DESeq2 analysis. A total of 26 ASVs were found to be significantly changed between the trichome samples of both genotypes. A total of 18 ASVs, including ASVs from the genus *Bacillus*, *Deinococcus*, *Acinetobacter*, *Paracoccus*, and *Sphingomonas*, were significantly increased in trichomes of genotype LA1777 compared to genotype LA4024. In contrast, 8 ASVs from the genus *Bacillus*, *Massilia*, *Caulobacter*, *Capnocytophaga*, *Pseudomonas*, *Pedobacter,* and *Luteimonas* were significantly increased in genotype LA4024 compared to genotype LA1777 (Fig. 5).

## Discussion

In the present study, the plant’s phyllosphere was explored in more spatial detail. We found differences in bacterial community structures and compositions in microhabitats that were so far not assessed separately. Our results highlighted an increased microbial diversity in association with tomato trichomes and distinct differences in bacterial occurrence compared to the rest of the leaf. Trichome samples harbor a unique and more diverse bacterial community than the surrounding trichome-free leaf surfaces. Even though the abundance of total bacteria (observed via qPCR) was not increased in trichome samples, a significantly higher alpha diversity (observed ASVs and Shannon index) was observed in trichomes compared to the other sample types. This indicates a distinct microbial community composition due to the specific microenvironment associated with glandular trichomes. Apart from micro and macro environmental conditions, we found indications that secondary molecules released in the trichomes could influence microbial compositions and attract specific microbes. It was previously found that glandular trichomes release a number of different liquid and volatile secondary metabolites for plant communication and especially defense [12, 18, 47, 48]. Moreover, tomato trichomes were shown to produce a genotype-specific molecule cocktail, which is important for resistance to herbivores [12] and a target for plant breeders [13]. These molecules, however can also attract a number of microorganisms, which was shown here and in previous studies using different microscopy techniques [20, 24]. This specific attraction could increase the accumulation of plant-beneficial bacteria close to potential entry points of pathogens [20]. Additionally, plant-associated microorganisms produce diverse bioactive metabolites including VOCs [49], which can contribute to the metabolic diversity in trichomes.

Even though plant trichomes were shown to play a key role in plant defense mechanisms, previous studies based on DGGE analyses of 16S rRNA genes came to the conclusion that *Arabidopsis* trichomes do not harbor a different microbial community compared to the whole leaf [11]. Using advanced high-throughput sequencing, we were able to assess bacterial communities in more detail and could show certain differences at taxonomic resolutions below phylum level. Detailed analysis showed especially an increased number of *Alphaproteobacteria*, including the genus *Sphingomonas*. This bacterial genus was previously shown to include typical phyllosphere microorganisms [50–52]. Additionally, it was identified as a plant-protecting bacterium, which is especially important for entry points at the plant surface [20, 52]. Plant phyllosphere pathogens, including *Clavibacter michiganensis*, as one of the most important bacterial pathogens in tomato, were shown to infect leaves via trichomes [21]. However, some wild tomato species including *S. habrochaites* LA2128, were found to be resistant to infections by *C. michiganensis*. Although the mechanism underlying resistance remains unclear, it is believed that the colonization of the pathogen is inhibited [22]. Furthermore, we have observed that relative abundances of *Actinobacteria* ASVs increased in trichomes. They are well-known as an important source of bioactive natural products [53, 54]. A recent study by Passari et al. (2019) demonstrated that inoculation of tomato plants with *Streptomyces thermocarboxydus* *(Actinobacteria)* had a positive effect on plant growth, conferred protection against plant pathogens as well as production of carotenoids, benzenoids and flavonoids [55]. Our results show that distinct plant-protecting taxa as well as enriched diversity might accumulate at this potential entry point for pathogens. This could be an indication for yet unexplored plant defense mechanisms, based on spatially enriched microorganisms in the plant phyllosphere.

We were further able to show that, apart from the overall observed trichome specific microbiomes, specific tomato genotypes were able to enrich different taxa on their trichomes. Genotype LA1777 was shown to support the colonization of its trichomes by a number of significantly different ASVs compared to genotype LA4024. These ASVs included taxa from the genus *Bacillus*, *Deinococcus*, *Massilia, Shingomonas*, and *Pseudomonas*. We hypothesize that this genotype-specific enrichment of trichome-colonizing microorganisms is due to changed microbial attraction, based on changed secondary metabolic profiles, previously studied in the used tomato genotype lines [12]. Bacteria, capable of metabolizing of the released metabolites could be attracted. *Pseudomonas aeruginosa*, for instance, was previously found to carry genetic pathways capable of catabolizing acyclic terpenes and leucine/isovalerate [56]. Further investigations, however, are necessary to confirm this hypothesis.

Our findings confirm that niches formed by a plant’s epidermal outgrowths are bacterial hotspots. This was already shown for root hairs, which act as a determinant of the microbiota thriving at the root-soil interface and ensuring exchange and protection [57]. The present work is the first to analyze trichomes as a separate phyllosphere microhabitat by means of a detailed microbiome assessment. The observed difference in microbial diversity associated with trichomes remained so far hidden due to dilution effects when the whole leaf is analyzed. Moreover, as plant-associated microorganisms are known to produce diverse secondary metabolites, analyzing trichomes-associated bacteria could lead to the discovery of new bioactive compounds in the future.

## Conclusions

Tomato glandular trichomes harbor a unique and genotype-specific microbiota of high microbial diversity. It is likely that the metabolites accumulated and released by glandular trichomes play a role in shaping a distinct microbial community and might lead to the enrichment of potentially plant-beneficial taxa and their metabolites at vulnerable entry points of phytopathogens. Our observations not only contribute to a better understanding of the plant microbiome, but also show the importance of micro-niches for structuring of bacterial communities within the plant phyllosphere. These findings could provide further clues for breeding of pathogen-resistant plants as well as so far unexploited resource to develop natural pathogen defense strategies.

## Supplementary information

**Additional file 1.**

**Additional file 2.**

**Additional file 3.**

## Data Availability

The dataset supporting the conclusions of this article is available in the European Nucleotide Archive (ENA) (https://www.ebi.ac.uk/ena) under the project number PRJEB37893.
