# Supplementary material for: Trichomes form genotype-specific microbial hotspots in the phyllosphere of tomato
Source: Environ Microbiome. 2020 Sep 17;15:17. doi: 10.1186/s40793-020-00364-9 (PMC8067393; doi:10.1186/s40793-020-00364-9)
Supplement: Supplementary file 1 — Additional file 1. [file 40793_2020_364_MOESM1_ESM.docx]

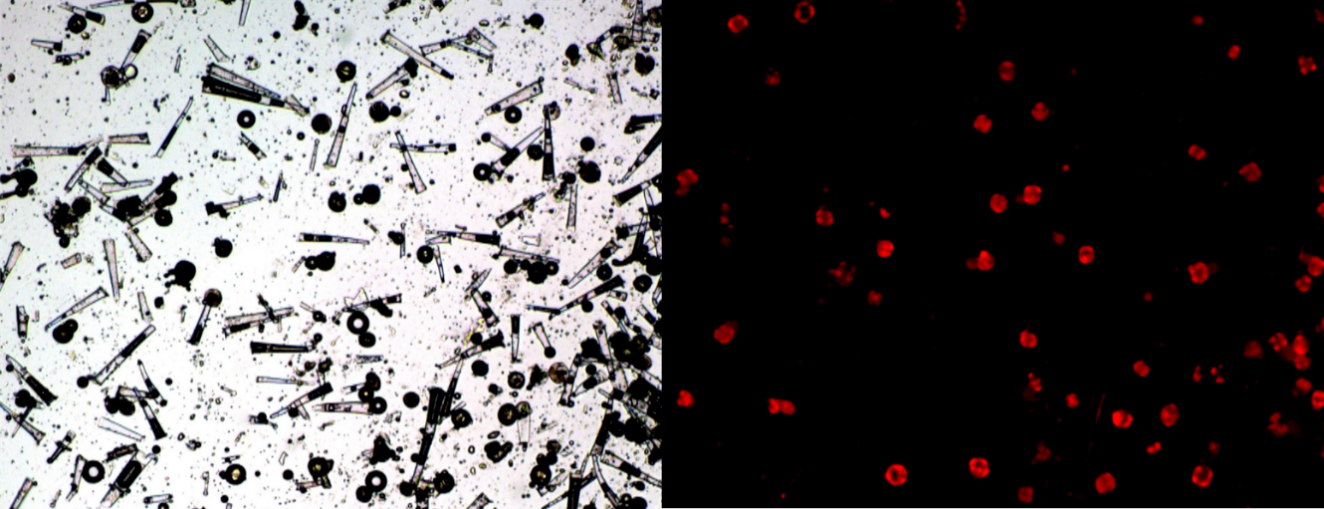

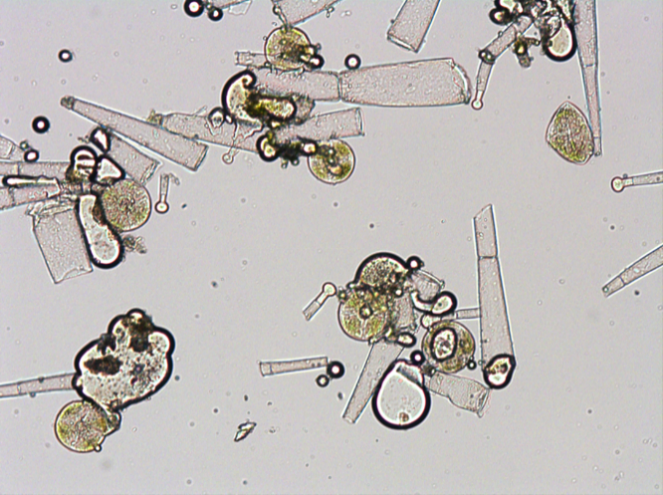


**A**

**C**


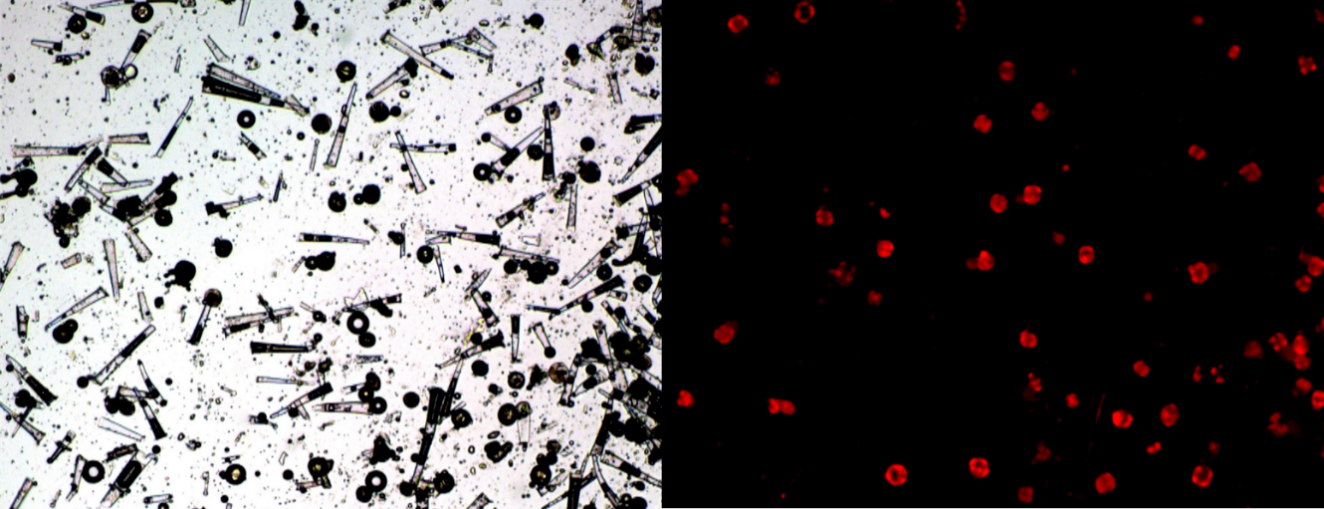


**B**

**Additional Figure:** **Microscopic observation during trichome isolation.** The panel (A) shows an overview of trichomes collected after harvest using a brush dipped in liquid nitrogen and sieving. The panel (B) is the same field but in autofluorescence of chlorophyll showing the heads of type VI trichomes. The panel (C) is a close-up view of the same sample. Pieces of trichomes stalks and trichome heads can be seen.
