# Supplementary material for: Trichomes form genotype-specific microbial hotspots in the phyllosphere of tomato
Source: Environ Microbiome. 2020 Sep 17;15:17. doi: 10.1186/s40793-020-00364-9 (PMC8067393; doi:10.1186/s40793-020-00364-9)
Supplement: Supplementary file 2 — Additional file 2. [file 40793_2020_364_MOESM2_ESM.docx]

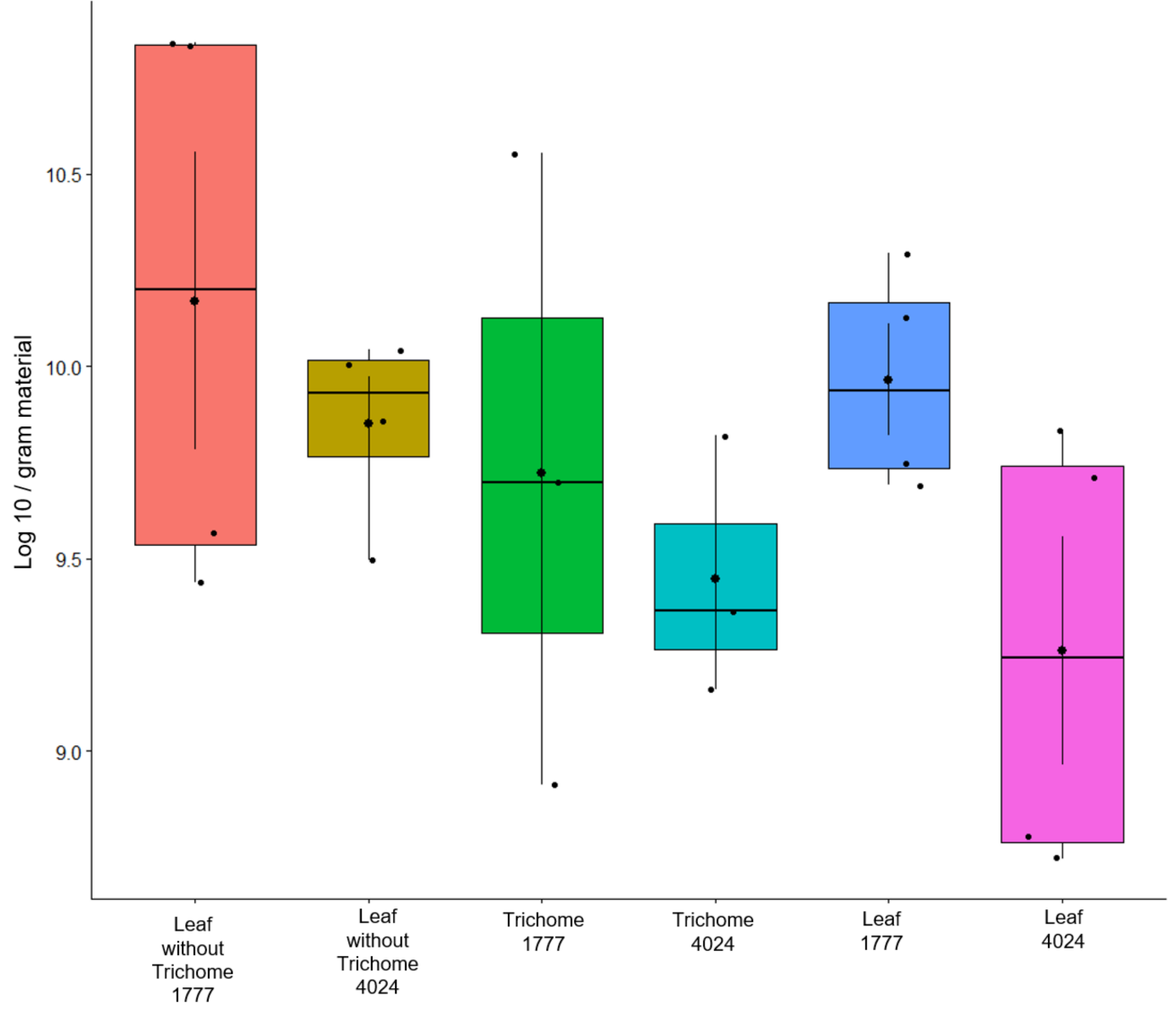


Figure: Real time qPCR of total bacterial numbers found in samples. Statistically significant differences were tested using Kruskal-Wallis, however, no statistical significance was found.
