# Supplementary material for: Trichomes form genotype-specific microbial hotspots in the phyllosphere of tomato
Source: Environ Microbiome. 2020 Sep 17;15:17. doi: 10.1186/s40793-020-00364-9 (PMC8067393; doi:10.1186/s40793-020-00364-9)
Supplement: Supplementary file 3 — Additional file 3. [file 40793_2020_364_MOESM3_ESM.docx]

Table: Read statistics of raw and filtered quality sequences of the amplicon dataset as well as observed alpha diversity of each sample.

| Sample ID | Sample type | Raw quality sequences | Filtered quality sequences | % non-target sequences | Observed ASVs | Shannon index |
| --- | --- | --- | --- | --- | --- | --- |
| A1 | Trichome LA4024 | 11858 | 1153 | 90.3 | 63 | 3.9 |
| A2 |  | 58036 | 8629 | 85.1 | 106 | 4.0 |
| A3 |  | 68466 | 5597 | 91.8 | 136 | 4.5 |
| A4 |  | 70000 | 7870 | 88.8 | 183 | 4.9 |
| A5 | Trichome LA1777 | 76554 | 10580 | 86.2 | 167 | 4.5 |
| A6 |  | 45174 | 16587 | 63.3 | 187 | 4.7 |
| A7 |  | 69024 | 7273 | 89.5 | 147 | 4.4 |
| A8 |  | 61741 | 13155 | 78.7 | 196 | 4.7 |
| B1 | Leaves  LA4024 | 37254 | 766 | 97.9 | 20 | 2.2 |
| B2 |  | 19621 | 660 | 96.6 | 15 | 2.5 |
| B3 |  | 30980 | 636 | 97.9 | 23 | 2.6 |
| B4 |  | 47546 | 723 | 98.5 | 12 | 1.8 |
| B5 | Leaves  LA1777 | 60143 | 2212 | 96.3 | 27 | 2.7 |
| B6 |  | 38013 | 3428 | 91 | 33 | 2.4 |
| B7 |  | 32523 | 1644 | 94.9 | 33 | 2.9 |
| B8 |  | 61330 | 691 | 98.9 | 22 | 2.5 |
| C1 | Leaves without trichome LA4024 | 79578 | 4374 | 94.5 | 60 | 3.7 |
| C2 |  | 18746 | 442 | 97.6 | 13 | 1.9 |
| C3 |  | 41677 | 1611 | 96.1 | 29 | 2.4 |
| C4 |  | 57330 | 2855 | 95 | 22 | 1.7 |
| C5 | Leaves without trichome LA1777 | 37744 | 3446 | 90.9 | 69 | 3.5 |
| C6 |  | 80842 | 6153 | 92.4 | 51 | 3.1 |
| C7 |  | 49089 | 2314 | 95.3 | 40 | 3.5 |
| C8 |  | 42447 | 1140 | 97.3 | 18 | 2.3 |
| **Average** | **-** | **49821.5** | **4330.7** | **91.9** | **69.7** | **3.2** |
